# Clinical and programmatic outcomes of HIV-exposed infants enrolled in care at geographically diverse clinics, 1997–2021: A cohort study

**DOI:** 10.1371/journal.pmed.1004089

**Published:** 2022-09-15

**Authors:** Andrew Edmonds, Ellen Brazier, Beverly S. Musick, Marcel Yotebieng, John Humphrey, Lisa L. Abuogi, Adebola Adedimeji, Olivia Keiser, Malango Msukwa, James G. Carlucci, Marcelle Maia, Jorge A. Pinto, Valériane Leroy, Mary-Ann Davies, Kara K. Wools-Kaloustian, on behalf of IeDEA

**Affiliations:** 1 Department of Epidemiology, Gillings School of Global Public Health, The University of North Carolina at Chapel Hill, Chapel Hill, North Carolina, United States of America; 2 Institute for Implementation Science in Population Health, Graduate School of Public Health and Health Policy, City University of New York, New York, New York, United States of America; 3 Department of Biostatistics and Health Data Science, Indiana University School of Medicine, Indianapolis, Indiana, United States of America; 4 Department of Medicine, Albert Einstein College of Medicine, Bronx, New York, United States of America; 5 Department of Medicine, Indiana University School of Medicine, Indianapolis, Indiana, United States of America; 6 Department of Pediatrics, University of Colorado, Denver, Aurora, Colorado, United States of America; 7 Department of Epidemiology & Population Health, Albert Einstein College of Medicine, Bronx, New York, United States of America; 8 Institute of Global Health, University of Geneva, Geneva, Switzerland; 9 Center for International Health, Education, and Biosecurity, University of Maryland, Lilongwe, Malawi; 10 Department of Pediatrics, Indiana University School of Medicine, Indianapolis, Indiana, United States of America; 11 Federal University of Minas Gerais, Belo Horizonte, Brazil; 12 Inserm, Université de Toulouse, CERPOP, Université Paul Sabatier, Toulouse, France; 13 University of Cape Town, Cape Town, South Africa; University of Southampton, UNITED KINGDOM

## Abstract

**Background:**

Although 1·3 million women with HIV give birth annually, care and outcomes for HIV-exposed infants remain incompletely understood. We analyzed programmatic and health indicators in a large, multidecade global dataset of linked mother–infant records from clinics and programs associated with the International epidemiology Databases to Evaluate AIDS (IeDEA) consortium.

**Methods and findings:**

HIV-exposed infants were eligible for this retrospective cohort analysis if enrolled at <18 months at 198 clinics in 10 countries across 5 IeDEA regions: East Africa (EA), Central Africa (CA), West Africa (WA), Southern Africa (SA), and the Caribbean, Central, and South America network (CCASAnet). We estimated cumulative incidences of DNA PCR testing, loss to follow-up (LTFU), HIV diagnosis, and death through 24 months of age using proportional subdistribution hazard models accounting for competing risks. Competing risks were transfer, care withdrawal, and confirmation of negative HIV status, along with LTFU and death, when not the outcome of interest. In CA and EA, we quantified associations between maternal/infant characteristics and each outcome. A total of 82,067 infants (47,300 EA, 10,699 CA, 6,503 WA, 15,770 SA, 1,795 CCASAnet) born from 1997 to 2021 were included. Maternal antiretroviral therapy (ART) use during pregnancy ranged from 65·6% (CCASAnet) to 89·5% (EA), with improvements in all regions over time. Twenty-four-month cumulative incidences varied widely across regions, ranging from 12·3% (95% confidence limit [CL], 11·2%,13·5%) in WA to 94·8% (95% CL, 94·6%,95·1%) in EA for DNA PCR testing; 56·2% (95% CL, 55·2%,57·1%) in EA to 98·5% (95% CL, 98·3%,98·7%) in WA for LTFU; 1·9% (95% CL, 1·6%,2·3%) in WA to 10·3% (95% CL, 9·7%,10·9%) in EA for HIV diagnosis; and 0·5% (95% CL, 0·2%,1·0%) in CCASAnet to 4·7% (95% CL, 4·4%,5·0%) in EA for death. Although infant retention did not improve, HIV diagnosis and death decreased over time, and in EA, the cumulative incidence of HIV diagnosis decreased substantially, declining to 2·9% (95% CL, 1·5%,5·4%) in 2020. Maternal ART was associated with decreased infant mortality (subdistribution hazard ratio [sdHR], 0·65; 95% CL, 0·47,0·91 in EA, and sdHR, 0·51; 95% CL, 0·36,0·74 in CA) and HIV diagnosis (sdHR, 0·40; 95% CL, 0·31,0·50 in EA, and sdHR, 0·41; 95% CL, 0·31,0·54 in CA). Study limitations include potential misclassification of outcomes in real-world service delivery data and possible nonrepresentativeness of IeDEA sites and the population of HIV-exposed infants they serve.

**Conclusions:**

While there was marked regional and temporal heterogeneity in clinical and programmatic outcomes, infant LTFU was high across all regions and time periods. Further efforts are needed to keep HIV-exposed infants in care to receive essential services to reduce HIV infection and mortality.

## Author summary

**Why was this study done?:**

- Clinical and programmatic outcomes among HIV-exposed infants are poorly documented, with gaps in information on receipt of antiretrovirals by infants and their mothers, early infant diagnosis and definitive diagnostic testing, anthropometric and developmental measures, and susceptibilities to mortality and loss to follow-up (LTFU).
- Previous evidence from both observational studies and clinical trials is largely limited to single-program or single-country reports with small sample sizes, lack of mother–infant linkages, or restricted geographical or temporal breadth.

**What did the researchers do and find?:**

- Pooling data from the International epidemiology Databases to Evaluate AIDS consortium (sub-Saharan Africa and Brazil) and the Umoyo+ project (Malawi) to create the largest global dataset of HIV-exposed infants to date, our study features >82,000 HIV-exposed infants, with >90% linked to maternal records, born over a 25-year period at 198 clinics providing routine HIV services in 10 countries—including 6 UNAIDS high HIV burden focus countries.
- Results from this retrospective cohort analysis provide robust, geographically broad insights on key clinical and programmatic outcomes across 5 regions, including the cumulative incidences of DNA PCR testing, LTFU, HIV diagnosis, and death through 24 months of age, accounting for relevant competing risks, while also utilizing mother–infant linkages to examine associations between these outcomes and maternal and infant characteristics.
- Notably, we found that while mortality and HIV diagnosis decreased over time globally, infant retention at 2 years did not exceed 60% in any period in any region.

**What do these findings mean?:**

- Although real-world service delivery data limitations include potential misclassification of outcomes and nonrepresentativeness of contributing sites, this study generated novel information on clinical characteristics and programmatic outcomes among HIV-exposed infants.
- While elucidating deficiencies in care that must be mitigated to deliver the package of medications and support necessary to assure that all such children remain uninfected, survive, and thrive, our analysis corroborated prior studies in highlighting unacceptably poor retention among those initially engaged in care.
- Our finding of widespread and persistent LTFU across disparate settings should motivate changes in policy and practice to improve patient retention, as maintaining infants in care will facilitate not only HIV testing and prevention, but also the provision of other services (primary care, nutrition, immunizations, etc.) essential for the achievement of long-term health.

## Introduction

Improved coverage of prevention of mother-to-child transmission (PMTCT) programs, along with the initiation of pregnant and breastfeeding women with HIV on lifelong treatment with antiretroviral therapy (ART) and the scale-up of immediate ART for all people with HIV [1], have contributed to dramatic declines in new pediatric HIV infections, from an estimated 520,000 annually in 2000 [2] to 150,000 in 2020 [3]. Nonetheless, there are an estimated 1·3 million pregnancies among women with HIV each year [2]. Available data indicate that compared to women without HIV, women with HIV are more likely to deliver infants who are preterm [4], are small for gestational age (SGA) [5], and have low birth weight (LBW) [5,6]—factors that are associated with mortality in HIV-exposed uninfected infants [7]. These infants have elevated risks of impaired growth (e.g., underweight) and neurodevelopment [8], lower respiratory tract infections and other comorbidities [9], and mortality [10]. To attain optimal health outcomes among children born to women with HIV and ensure that they remain “alive, HIV free, and thriving [11],” retention in care and access to a range of services are critical [12]. Such services include nutritional support, prophylactic antiretrovirals (ARVs) and cotrimoxazole, and regular HIV testing from early infant diagnosis (EID) through definitive diagnosis at 18 months of age or 3 months after breastfeeding cessation, whichever is later [13].

While there is a growing population of HIV-exposed infants worldwide [2], documentation of their clinical and programmatic outcomes is limited, with much of the extant knowledge based on modeling estimates and smaller to medium studies with restricted geographical or temporal breadth [11,14–16]. In general, linkages between maternal and HIV-exposed infant data are uncommon in clinic settings [12], which limits the ability to systematically examine the impacts of pregnancy and delivery characteristics on subsequent pediatric outcomes. In addition, loss to follow-up (LTFU) of HIV-exposed infants is high, with meta-analyses reporting attrition of 34% [17] at 3 months and 39% [18] at 18 months of age, leading to gaps in DNA PCR testing [3] and low ascertainment of HIV status.

To address knowledge gaps and provide geographical context, we analyzed a unique and sizeable multidecade dataset of linked mother–infant records from multiple regions of the International epidemiology Databases to Evaluate AIDS (IeDEA) consortium. Our primary outcomes of interest were the cumulative incidences of DNA PCR testing, LTFU, HIV diagnosis, and death through 24 months of age among HIV-exposed infants, focusing on variations by region and calendar time, as local HIV epidemics, sociocultural contexts, and programmatic environments were heterogenous in the periods and areas represented in this dataset. Along with describing characteristics of HIV-exposed infants and their mothers, we aimed to assess associations of ART during pregnancy, preterm birth (PTB), SGA, LBW, and calendar time with the primary outcomes.

## Methods

### Study population

Data for this retrospective cohort analysis were drawn from IeDEA, an international research consortium that pools and harmonizes data of patients receiving routine HIV care in 7 regional cohorts (Asia-Pacific [AP], Central Africa [CA], East Africa [EA], Southern Africa [SA], West Africa [WA], the Caribbean, Central and South America network for HIV epidemiology [CCASAnet], and the North American AIDS Cohort Collaboration on Research and Design [NA-ACCORD]) [19], as well as from the Umoyo+ project in Malawi [20]. Infants were eligible if they were documented as having a mother with HIV and if they enrolled in care (i.e., first database entry) at <18 months of age. Infants enrolled in ART cohorts (i.e., sites that provide care only to those with confirmed HIV) were excluded, as were IeDEA cohorts with no eligible infants (AP and NA-ACCORD). Data from the mothers of eligible infants were also included. The concept proposal underlying this analysis is included as Supporting information (S1 Concept Proposal).

### Measures

We defined measures as follows. PTB: <37 weeks of gestation. SGA: <10th percentile (sex-specific) for birthweight, as calculated by the Fenton 2013 Preterm Growth Chart [21]. LBW: <2,500 grams. Underweight at 6 weeks: weight-for-age Z-score <−2, as calculated by sex-specific WHO child growth standards [22], using the closest available weight measurement within 14 days to 6 weeks of age. HIV diagnosis: positive DNA PCR or RNA viral load at any age, or positive serology at >18 months of age. Confirmation of negative HIV status: negative DNA PCR, RNA viral load, or serology at >18 months of age, in the absence of prior HIV diagnosis. LTFU: final attended clinic visit >6 months prior to program-specific database closure date and no documentation of death, transfer, or care withdrawal, with censoring on the final visit date [23]. Calendar time (year of infant birth): pre-2010 (i.e., years prior to the Option A and Option B approaches for PMTCT), 2010 to 2013 (Options A and B), 2014 to 2015 (Option B+), and post-2015 (“Treat All”), categorized to correspond approximately with 4 WHO HIV prevention and treatment guideline periods [1,13].

### Statistical analysis

Descriptive statistics, including proportions, medians, and interquartile ranges (IQRs), were used to characterize HIV-exposed infants, their mothers, and outcomes of interest for each region. Given the heterogeneity of source data, certain variables (e.g., gestational age at birth, DNA PCR testing) were unavailable in selected regions, often because the data are not recorded during patient care, and in WA, infants generally could not be linked to mothers. Accordingly, records missing data were excluded when calculating percentages.

Cumulative incidence functions through 24 months of age, accounting for competing risks, were estimated for the 4 primary outcomes (DNA PCR testing, LTFU, HIV diagnosis, and death) via proportional subdistribution hazard models [24], stratified by region and by calendar time within each region. Competing risks were transfer, care withdrawal, and confirmation of negative HIV status, along with LTFU and death, when not the outcome of interest. In sensitivity analyses aimed at approximating estimates among HIV-exposed uninfected infants rather than all HIV-exposed infants, we included HIV diagnosis as an additional competing risk (for outcomes besides HIV diagnosis). All cumulative incidence estimates accounted for left truncation (i.e., infants were considered at risk for outcomes beginning on the date of program enrollment rather than beginning on the date of birth). To provide insight on early testing, we also reported the cumulative incidences of DNA PCR testing by 2 months of age.

In EA and CA, where data were sufficiently complete and sample size was large enough, multivariable proportional subdistribution hazards models were used to quantify associations of ART during pregnancy, PTB, SGA, LBW, and calendar time with the primary outcomes. Complete case analysis was used because multiple imputation may lead to bias when data are missing not at random [25] and because our dataset had a limited number of variables for prediction. We assumed data were missing not at random, as they were extracted from heterogenous medical records and databases across diverse settings, where missing values may reflect differences in health information management systems and patient medical charts, as well as differences in delivery of pregnancy and postnatal care, rather than patient characteristics. Associations between missingness of variables, and trends in data missingness over time, are detailed in the Supporting information (S1 Missingness). The missingness analysis was performed in response to peer review comments.

Methods for mother–infant linkages varied by region. Except in SA, individuals had unique identifiers, with pre- and postnatal data generally contained in the same database (whether from an HIV care and treatment center, or from a pregnancy register at a maternal and child health [MCH] clinic). In SA, probabilistic record linkage methods, relying on demographic characteristics such as date of birth, sex, location, and names, were used. Data abstracted from patient medical records or extracted from electronic medical records were deidentified and cleaned by local and regional data managers prior to transfer for use in analyses. Approvals for data collection and contribution to IeDEA were obtained by participating regions from relevant institutional review boards and ethics committees, with informed consent sought where required by national ethics committees. The analysis was approved by the institutional review board at the University of North Carolina at Chapel Hill (18–3313) and by IeDEA’s Executive Committee. Sharing of Umoyo+ data for this analysis was approved by the Malawi National Health Sciences Research Committee (MED/4/36c). All analyses were completed in SAS version 9·4 (SAS Institute, Cary, NC, USA). This study is reported as per the REporting of studies Conducted using Observational Routinely-collected Data (RECORD) guideline (S1 STROBE Checklist).

## Results

In total, 82,067 HIV-exposed infants born from 1997 to 2021 were eligible and included, with 92·4% linked to maternal data (i.e., at least 1 maternal variable available). The numbers and types of study sites (i.e., HIV care and treatment center, or MCH clinic), along with numbers and birth year ranges of infants, are detailed by region and country in Table 1. Infants received care at 198 sites across 10 countries in 5 IeDEA regions: Kenya in EA (*n* = 55); Republic of Congo, Democratic Republic of Congo (DRC), and Burundi in CA (*n* = 113); Côte d’Ivoire, Ghana, Togo, and Benin in WA (*n* = 8); Malawi in SA (*n* = 21); and Brazil in CCASAnet (*n* = 1). The largest numbers of infants were from the EA (*n* = 47,300) and SA (*n* = 15,770) regions of IeDEA. Data from most countries included infants born throughout the full study period, with some exceptions (e.g., Umoyo+ in Malawi contributed data from infants born between 2010 and 2015 only). MCH clinics in the sample were in EA (*n* = 16) and CA (*n* = 106).

**Table 1:** HIV-exposed infants and sites in the IeDEA consortium and Umoyo+, by region and country, 1997–2021.

Region	Country	Number	Linked to Mother*	Sites	Program or Site	Birth Years
East Africa	Kenya	47,300	100%	55	AMPATH, FACES	2001–2021
Central Africa	DRC	2,570	100%	2	Kalembe Lembe, Bomoi	2003–2018
	DRC	3,780	100%	106	Various MCH clinics	2014–2019
	Burundi	1,922	100%	3	Association Nationale de Soutien aux Séropositifs et Malade du Sida, Centre Hospitalo-Universitaire de Kamenge, l’Hôpital Prince Régent Charles	2005–2020
	Republic of Congo	2,427	100%	2	Brazzaville, Pointe-Noire	2001–2019
West Africa	Côte d’Ivoire	2,676	8·9%	5	Centre de Prise en Charge de Recherche et de Formation, Centre Hospitalier Universitaire de Cocody, Centre Hospitalier Universitaire de Yopougon, Centre Intégré de Recherche Bioclinique d’Abidjan, MTCT-Plus	2004–2018
	Benin	1,355	0%	1	Centre National Hospitalier Universitaire, Cotonou	2002–2017
	Ghana	1,237	0%	1	Korle-Bu Teaching Hospital	2004–2018
	Togo	1,235	0%	1	Centre Hospitalier Universitaire de Tokoin	2011–2017
Southern Africa	Malawi	15,770	100%	21	Umoyo+	2010–2015
CCASAnet	Brazil	1,795	100%	1	Universidade Federal de Minas Gerais	1997–2017
**Total**		**82,067**	**92·4%**	**198**		**1997–2021**

AMPATH, Academic Model Providing Access to Healthcare; CCASAnet, Central and South America network for HIV epidemiology; DRC, Democratic Republic of Congo; FACES, Family AIDS Care and Education Services; IeDEA, International epidemiology Databases to Evaluate AIDS; MCH, maternal and child health.

*At least 1 maternal variable available.

All sites were HIV care and treatment centers except for those noted as MCH clinics. Of the 55 HIV care and treatment centers in East Africa, 16 had an affiliated MCH clinic.

Characteristics of mothers and infants, along with outcomes at delivery and over the subsequent 24-month period, are detailed in Table 2. Median maternal age at delivery was approximately 30 years in each region with available data. Between 80·1% (CCASAnet) and 97·9% (WA) of mothers received any ARVs during pregnancy, with 65·6% (CCASAnet) to 89·5% (EA) on ART prior to delivery; maternal ARV/ART uptake improved over time across regions. Just over half of infants were female (50·9%), ranging from 47·8% in CCASAnet to 58·6% in CA. Infants entered HIV care early, with the highest median age at care enrollment in SA (1·8 months; IQR, 1·5 to 4·5). PTB was more common in CCASAnet and CA at 17·9% and 19·2%, respectively, than in EA (5·5%). CA had the highest percentage of SGA infants at 32·1%, versus 25·1% in CCASAnet and 18·9% in EA. LBW ranged from 9·7% in EA to 21·4% in CCASAnet, with underweight at 6 weeks ranging from 2·2% in SA to 21·7% in WA. Receipt of ARV and cotrimoxazole prophylaxis by infants varied substantially by region. The highest proportion of infants receiving ARVs (e.g., extended nevirapine) was 92·0% in CCASAnet, followed by SA (65·2%), EA (58·6%), CA (50·9%), and WA (17·1%). Higher percentages of infants received cotrimoxazole in EA (94·8%) and SA (86·7%) than in CA (56·1%) and WA (32·9%). While there were small increases in receipt of ARVs and cotrimoxazole by infants in each region, there were no consistent temporal trends over time in these programmatic measures or in infant anthropometric and developmental measures (PTB, LBW, and SGA). The proportion of infants documented as alive, in care, and HIV-free at 18 months ranged from 34·4% in EA to 2·5% in WA and did not improve over time.

**Table 2:** Clinical and programmatic outcomes of HIV-exposed infants and their mothers in the IeDEA consortium and Umoyo+, by region, 1997–2021.

	East Africa (*n* = 47,300)	Central Africa (*n* = 10,699)	West Africa (*n* = 6,503)	Southern Africa (*n* = 15,770)	CCASAnet (*n* = 1,795)
Maternal age (years) at delivery, median (IQR)	29·1 (24·9–33·6) [*n* = 35,439]	31·5 (27·0–35·7) [*n* = 10,274]	··	··	28·9 (24·6–33·9) [*n* = 1,137]
ARVs during pregnancy	25,822 (91·7) [*n* = 28,147]	7,843 (84·9) [*n* = 9,243]	234 (97·9) [*n* = 239]	12,644 (80·2) [*n* = 15,770]	1,438 (80·1) [*n* = 1,795]
pre-2010	6,455 (85·8)	742 (60·0)	··	··	1,002 (74·5)
2010–2013	9,624 (90·8)	1,442 (76·0)	87 (96·7)	10,981 (78·9)	222 (95·7)
2014–2015	4,634 (95·8)	1,551 (91·1)	84 (97·7)	1,663 (90·0)	100 (98·0)
post-2015	5,109 (98·5)	4,108 (93·2)	63 (100·0)	··	114 (98·3)
≥3 ARVs during pregnancy	25,195 (89·5) [*n* = 28,147]	7,627 (82·5) [*n* = 9,243]	207 (86·6) [*n* = 239]	11,337 (71·9) [*n* = 15,770]	1,178 (65·6) [*n* = 1,795]
pre-2010	6,403 (85·1)	577 (46·7)	··	··	748 (55·6)
2010–2013	9,199 (95·6)	1,432 (75·4)	78 (86·7)	9,767 (70·2)	218 (94·0)
2014–2015	4,484 (92·7)	1,537 (90·3)	74 (86·0)	1,570 (85·0)	99 (97·1)
post-2015	5,109 (98·5)	4,081 (92·6)	55 (87·3)	··	113 (97·4)
Infant year of birth	[*n* = 47,300]	[*n* = 10,699]	[*n* = 6,503]	[*n* = 15,770]	[*n* = 1,795]
pre-2010	15,273 (32·3)	1,755 (16·4)	1,481 (22·8)	0 (0·0)	1,345 (74·9)
2010–2013	17,197 (36·4)	2,415 (22·6)	2,576 (39·6)	13,923 (88·3)	232 (12·9)
2014–2015	7,299 (15·4)	1,846 (17·3)	1,357 (20·9)	1,847 (11·7)	102 (5·7)
post-2015	7,531 (15·9)	4,683 (43·8)	1,089 (16·7)	0 (0·0)	116 (6·5)
Female sex	24,358 (51·5) [*n* = 47,300]	5,792 (58·6) [*n* = 9,891]	3,167 (48·7) [*n* = 6,503]	7,586 (50·9) [*n* = 14,901]	858 (47·9) [*n* = 1,790]
Infant age (months) at care enrollment, median (IQR)	1·4 (0·6–2·6) [*n* = 47,300]	0·0 (0·0–0·3) [*n* = 10,699]	0·9 (0·1–2·0) [*n* = 6,503]	1·8 (1·5–4·5) [*n* = 15,770]	0·0 (0·0–0·0) [*n* = 1,795]
pre-2010	1·6 (0·7–5·3)	0·5 (0·0–1·5)	1·5 (0·5–3·2)	··	0·0 (0·0–0·0)
2010–2013	1·0 (0·4–2·4)	0·5 (0·0–0·8)	0·8 (0·0–2·0)	2·0 (1·5–5·2)	0·0 (0·0–0·0)
2014–2015	1·3 (0·6–1·6)	0·0 (0·0–0·0)	0·3 (0·0–1·6)	1·6 (1·4–1·9)	0·0 (0·0–0·0)
post-2015	1·4 (1·3–1·6)	0·0 (0·0–0·0)	0·5 (0·0–1·7)	··	0·0 (0·0–0·0)
PTB (<37 weeks)	1,422 (5·5) [*n* = 25,840]	1,815 (19·2) [*n* = 9,435]	··	··	147 (17·9) [*n* = 823]
pre-2010	315 (4·6)	195 (13·2)	··	··	75 (19·2)
2010–2013	527 (5·4)	185 (8·7)	··	··	37 (16·4)
2014–2015	319 (7·4)	344 (20·6)	··	··	21 (22·1)
post-2015	261 (5·4)	1,091 (26·2)	··	··	14 (12·4)
LBW (<2,500 grams)	2,871 (9·7) [*n* = 29,620]	1,314 (13·5) [*n* = 9,703]	710 (20·6) [*n* = 3,441]	1,354 (12·2) [*n* = 11,066]	380 (21·4) [*n* = 1,776]
pre-2010	1,000 (12·5)	231 (15·5)	70 (23·5)	··	281 (21·1)
2010–2013	1,054 (9·5)	278 (12·5)	282 (19·6)	1,205 (12·4)	43 (18·7)
2014–2015	430 (7·9)	248 (14·6)	210 (21·9)	139 (11·1)	31 (30·4)
post-2015	387 (7·7)	557 (13·0)	148 (19·9)	··	25 (21·9)
SGA	3,457 (18·9) [*n* = 18,251]	2,631 (32·1) [*n* = 8,195]	··	··	193 (25·1) [*n* = 770]
pre-2010	950 (21·6)	478 (36·2)	··	··	91 (25·0)
2010–2013	1,442 (20·3)	699 (36·3)	··	··	52 (24·4)
2014–2015	565 (16·7)	490 (33·3)	··	··	26 (29·9)
post-2015	500 (14·9)	964 (27·7)	··	··	24 (22·6)
Underweight at 6 weeks*	3,346 (12·2) [*n* = 27,438]	689 (19·7) [*n* = 3,493]	281 (21·7) [*n* = 1,295]	109 (2·2) [*n* = 5,042]	198 (19·1) [*n* = 1,039]
pre-2010	1,256 (17·5)	89 (17·8)	53 (19·2)	··	159 (19·8)
2010–2013	1,148 (11·2)	140 (15·2)	103 (23·5)	93 (2·2)	20 (14·7)
2014–2015	449 (9·2)	113 (26·2)	66 (20·9)	16 (1·9)	11 (17·2)
post-2015	493 (9·5)	347 (21·1)	59 (22·3)	··	8 (22·2)
Infant cotrimoxazole at <24 months	44,836 (94·8) [*n* = 47,300]	6,005 (56·1) [*n* = 10,699]	2,138 (32·9) [*n* = 6,503]	13,675 (86·7) [*n* = 15,770]	··
pre-2010	14,204 (93·0)	956 (54·5)	507 (34·2)	··	··
2010–2013	16,259 (94·5)	1,267 (52·5)	803 (31·2)	12,059 (86·6)	··
2014–2015	6,941 (95·1)	817 (44·3)	425 (31·3)	1,616 (87·5)	··
post-2015	7,432 (98·7)	2,965 (63·3)	403 (37·0)	··	··
Infant ARV prophylaxis at <24 months	27,716 (58·6) [*n* = 47,300]	5,441 (50·9) [*n* = 10,699]	1,113 (17·1) [*n* = 6,503]	10,287 (65·2) [*n* = 15,770]	1,652 (92·0) [*n* = 1,795]
pre-2010	3,276 (21·4)	818 (46·6)	259 (17·5)	··	1,234 (91·7)
2010–2013	12,530 (72·9)	1,082 (44·8)	379 (14·7)	9,012 (64·7)	221 (95·3)
2014–2015	6,308 (86·4)	824 (44·6)	243 (17·9)	1,275 (69·0)	87 (85·3)
post-2015	5,602 (74·4)	2,717 (58·0)	232 (21·3)	··	110 (94·8)
Duration of follow-up (months), median (IQR)^$^	15·7 (6·7–19·2) [*n* = 47,300]	6·8 (1·3–15·8) [*n* = 10,699]	0·5 (0·0–7·5) [*n* = 6,503]	8·3 (1·4–15·4) [*n* = 15,770]	15·1 (9·2–19·9) [*n* = 1,795]
pre-2010	15·9 (6·7–19·3)	5·0 (1·0–11·7)	2·1 (0·0–12·4)	··	15·2 (9·7–20·3)
2010–2013	17·1 (8·3–20·3)	10·8 (1·9–17·6)	0·0 (0·0–5·8)	9·4 (1·8–16·3)	17·4 (13·7–22·1)
2014–2015	13·8 (5·5–17·6)	6·9 (0·2–18·0)	0·0 (0·0–5·7)	3·1 (1·0–7·9)	15·9 (12·6–19·0)
post-2015	12·2 (5·7–17·1)	5·7 (1·1–13·1)	0·9 (0·0–5·9)	··	5·5 (4·2–10·1)
Alive, in care, and HIV-free at 18 months^#^	14,912 (34·4) [*n* = 43,369]	1,017 (11·0) [*n* = 9,276]	162 (2·7) [*n* = 5,907]	2,274 (17·4) [*n* = 13,056]	347 (19·3) [*n* = 1,795]
pre-2010	6,502 (42·8)	136 (7·7)	38 (2·6)	··	288 (21·4)
2010–2013	6,088 (36·5)	259 (11·5)	73 (2·8)	2,274 (18·4)	48 (20·7)
2014–2015	847 (12·4)	183 (9·9)	43 (3·2)	0 (0·0)	9 (8·8)
post-2015	1,475 (31·8)	439 (12·8)	8 (1·6)	··	2 (1·7)

*Weight-for-age Z-score <−2, as calculated by sex-specific WHO child growth standards.

^$^Through 24 months of age.

^#^Among infants 18 months of age as of database closure date.

ARV, antiretroviral; CCASAnet, Central and South America network for HIV epidemiology; IeDEA, International epidemiology Databases to Evaluate AIDS; IQR, interquartile range; LBW, low birth weight; PTB, preterm birth; SGA, small for gestational age.

All results are *n* (%) unless otherwise noted. Percentages and medians calculated using denominators of individuals with nonmissing data (e.g., gestational age at birth was not available for some infants, and ARV receipt during pregnancy was not available for some mothers). If data for a specific variable were not available in a region, this is denoted by “··” in the table.

**Fig 1:**
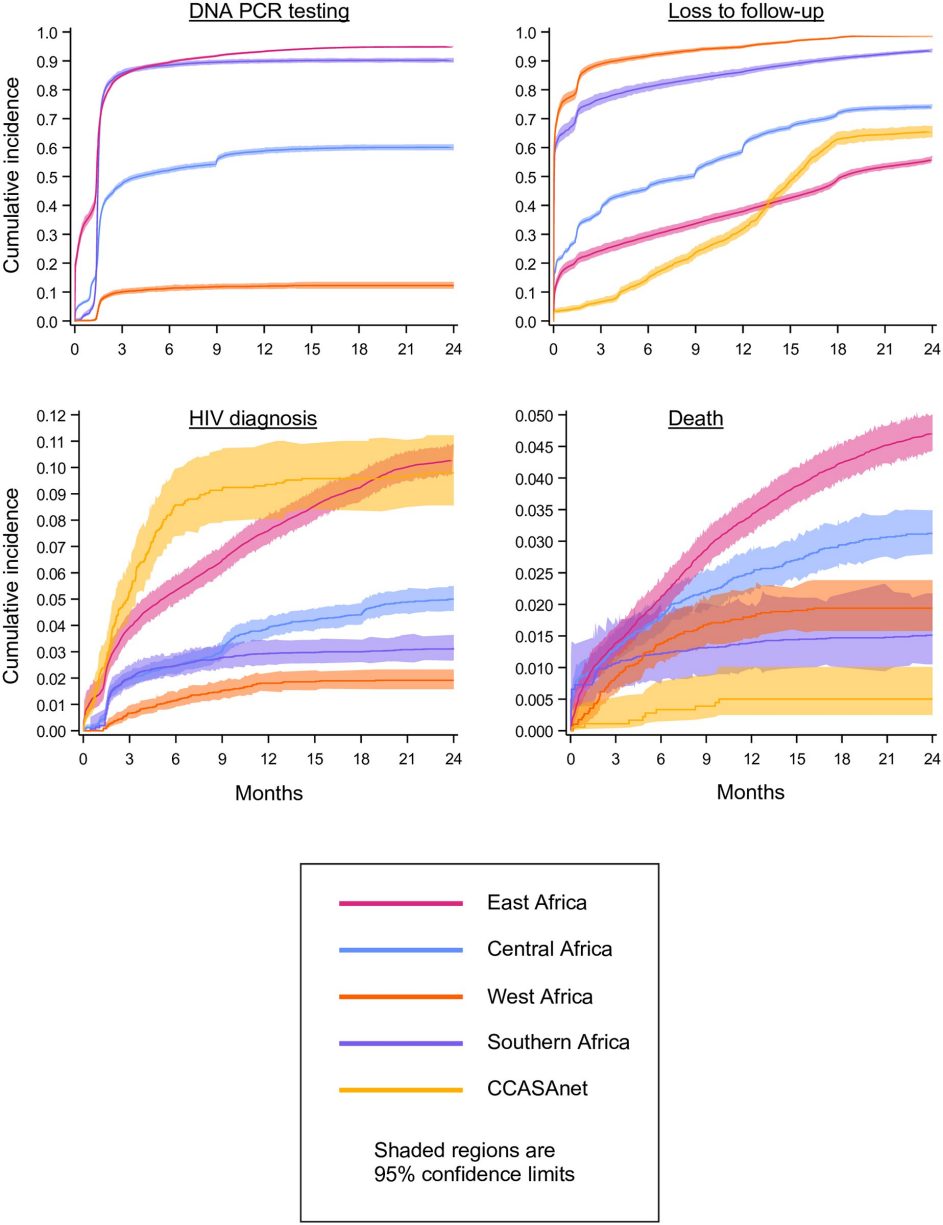
Cumulative incidences of DNA PCR testing, LTFU, HIV diagnosis, and death through 24 months of age among HIV-exposed infants in the IeDEA consortium and Umoyo+, by region, 1997–2020. CCASAnet, Caribbean, Central, and South America network for HIV epidemiology; IeDEA, International epidemiology Databases to Evaluate AIDS; LTFU, loss to follow-up.

Cumulative incidences of each primary outcome through 24 months of age across all time periods are plotted in Fig 1, by region. The cumulative incidence of DNA PCR testing at 2 months was 80·7% (95% confidence limit [CL], 79·5%,82·0%) in SA, 78·0% (95% CL, 77·2%,78·8%) in EA, 41·9% (95% CL, 40·9%,42·8%) in CA, and 8·5% (95% CL, 7·6%,9·5%) in WA. The pattern was similar at 24 months, with DNA PCR testing above 90% in EA (94·8%; 95% CL, 94·6%,95·1%) and SA (90·2%; 95% CL, 89·4%,91·0%), 60·1% (95% CL, 59·1%,61·1%) in CA, and 12·3% (95% CL, 11·2%,13·5%) in WA. Despite variability in DNA PCR testing, the median age at first test was <2 months in all regions. LTFU was universally high, ranging from 56·2% (95% CL, 55·2%,57·1%) in EA to 93·6% in SA (95% CL, 92·9%,94·2%) and 98·5% in WA (95% CL, 98·3%,98·7%). The 24-month cumulative incidence of HIV diagnosis ranged from 1·9% (95% CL, 1·6%,2·3%) in WA to 10·3% (95% CL, 9·7%,10·9%) in EA, with the median age at diagnosis highest in EA (8·4 months; IQR, 3·2 to 14·1) and lowest in CCASAnet (2·7 months; IQR, 1·4 to 4·6). Cumulative incidence functions revealed that HIV diagnoses occurred throughout follow-up, suggesting missed opportunities for diagnosis among infants not retained in care beyond the EID period. The 24-month cumulative incidence of death ranged from 4·7% (95% CL, 4·4%,5·0%) in EA to 0·5% (95% CL, 0·2%,1·0%) in CCASAnet. Estimates from sensitivity analyses with HIV diagnosis as an additional competing risk returned similar, slightly lower estimates of cumulative incidence (S1 Table).

**Fig 2:**
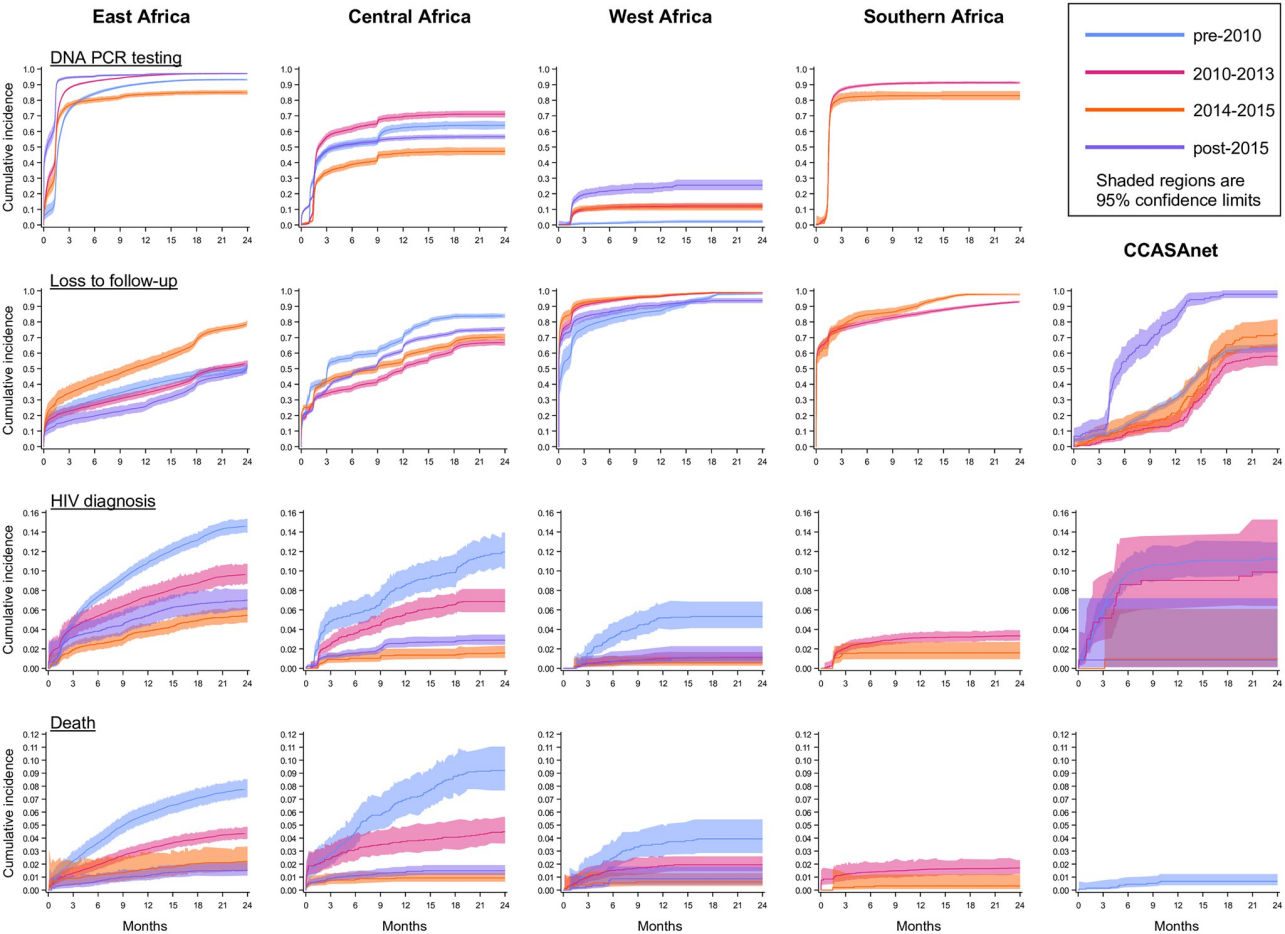
Cumulative incidences of DNA PCR testing, LTFU, HIV diagnosis, and death through 24 months of age among HIV-exposed infants in the IeDEA consortium and Umoyo+, by region and time period, 1997–2020. CCASAnet, Caribbean, Central, and South America network for HIV epidemiology; IeDEA, International epidemiology Databases to Evaluate AIDS; LTFU, loss to follow-up.

Fig 2 depicts the cumulative incidences of our primary outcomes of interest by region and time period, with cumulative incidences of each outcome shown by birth year for EA only in S1 Fig. DNA PCR testing was low in WA across all time periods but improved post-2010; levels were consistently moderate in CA, with lower DNA PCR testing in more recent time periods. In EA and SA, DNA PCR testing was consistently high (>80%) across time periods. In CCASAnet and CA, LTFU was lower in more recent time periods than pre-2010, but incremental improvements were not evident. In EA, WA, and SA, LTFU did not lessen over time. In EA, CA, and SA, HIV diagnosis and death were lower in more recent time periods than in earlier time periods; in WA, these estimates were lower post-2010. In EA, where 84·1% of infants in our sample were born before 2016, HIV diagnosis decreased substantially over time, declining to 2·9% (95% CL, 1·5%,5·4%) in 2020 (also shown in S1 Fig).

Associations of ART during pregnancy, PTB, SGA, LBW, and calendar time with the primary outcomes are detailed in Table 3 for EA and CA. In EA, ART during pregnancy was associated with increased DNA PCR testing (subdistribution hazard ratio [sdHR], 1·37; 95% CL, 1·26,1·49) and with decreased LTFU (sdHR, 0·57; 95% CL, 0·53,0·63), HIV diagnosis (sdHR, 0·40; 95% CL, 0·31,0·50), and infant death (sdHR, 0·65; 95% CL, 0·47,0·91). Additionally, LBW was associated with decreased DNA PCR testing (sdHR, 0·91; 95% CL, 0·84,0·99) and increased death (sdHR, 2·04; 95% CL, 1·42,2·94). In CA, ART during pregnancy was also associated with decreased HIV diagnosis (sdHR, 0·41; 95% CL, 0·31,0·54) and infant death (sdHR, 0·51; 95% CL, 0·36,0·74) but was associated with decreased DNA PCR testing (sdHR, 0·91; 95% CL, 0·84,0·99). Like in EA, LBW was associated with increased death (sdHR, 3·15; 95% CL, 1·96,5·09) and decreased DNA PCR testing (sdHR, 0·86; 95% CL, 0·77,0·96).

**Table 3:** Associations of maternal and infant characteristics with DNA PCR testing, LTFU, HIV diagnosis, and death among HIV-exposed infants in the EA and CA regions of the IeDEA consortium, 2001–2021.

			EA		CA	
Maternal or infant characteristic		Outcome	sdHR	95% CL	sdHR	95% CL
ART during pregnancy		DNA PCR testing	1·37	1·26,1·49	0·91	0·84,0·99
		LTFU	0·57	0·53,0·63	1·00	0·92,1·08
		HIV diagnosis	0·40	0·31,0·50	0·41	0·31,0·54
		Death	0·65	0·47,0·91	0·51	0·36,0·74
LBW		DNA PCR testing	0·91	0·84,0·99	0·86	0·77,0·96
		LTFU	1·09	0·98,1·22	1·07	0·97,1·18
		HIV diagnosis	1·25	0·88,1·76	1·23	0·81,1·87
		Death	2·04	1·42,2·94	3·15	1·96,5·09
PTB		DNA PCR testing	0·85	0·79,0·92	1·04	0·95,1·14
		LTFU	0·91	0·82,1·00	1·08	1·00,1·17
		HIV diagnosis	1·02	0·71,1·49	1·43	0·97,2·09
		Death	1·16	0·75,1·78	1·03	0·63,1·66
SGA		DNA PCR testing	1·12	1·06,1·18	1·03	0·96,1·11
		LTFU	0·92	0·85,1·00	1·01	0·94,1·07
		HIV diagnosis	0·96	0·74,1·27	1·22	0·89,1·67
		Death	1·10	0·81,1·52	0·78	0·49,1·23
Calendar time (infant year of birth)	pre-2010	DNA PCR testing	ref.		ref.	
	2010–2013		1·09	1·04,1·13	1·03	0·93,1·14
	2014–2015		0·57	0·53,0·60	0·59	0·53,0·66
	post-2015		0·82	0·77,0·88	0·94	0·85,1·04
	pre-2010	LTFU	ref.		ref.	
	2010–2013		1·26	1·18,1·36	0·51	0·47,0·56
	2014–2015		3·19	2·96,3·44	0·59	0·54,0·65
	post-2015		1·40	1·27,1·53	0·65	0·60,0·71
	pre-2010	HIV diagnosis	ref.		ref.	
	2010–2013		0·55	0·46,0·67	0·49	0·33,0·73
	2014–2015		0·33	0·25,0·45	0·26	0·15,0·44
	post-2015		0·31	0·22,0·44	0·56	0·40,0·77
	pre-2010	Death	ref.		ref.	
	2010–2013		0·63	0·51,0·79	0·64	0·41,1·00
	2014–2015		0·29	0·20,0·42	0·20	0·10,0·38
	post-2015		0·22	0·14,0·36	0·32	0·20,0·50

ART, antiretroviral therapy; CA, Central Africa; CL, confidence limit; EA, East Africa; IeDEA, International epidemiology Databases to Evaluate AIDS; LBW, low birth weight; LTFU, loss to follow-up; PTB, preterm birth; sdHR, subdistribution hazard ratio; SGA, small for gestational age.

Estimates are based on infants who had data for all 5 characteristics (*n* = 18,251 in EA and *n* = 7,373 in CA), with the sdHR for a given characteristic estimated using a multivariable model including the other 4 characteristics in the table.

In EA, compared with pre-2010, more recent time periods were associated with decreased DNA PCR testing, HIV diagnosis, and death, along with increased LTFU (e.g., post-2015 sdHR, 1·40; 95% CL, 1·27,1·53). Similarly in CA, later time periods were associated with decreased HIV diagnosis and death, along with decreased LTFU; however, these decreases were not monotonic.

## Discussion

Findings from this analysis of a multidecade global dataset of linked mother–infant records, including data from 6 of the 21 UNAIDS Start Free, Stay Free, AIDS Free focus countries in sub-Saharan Africa [3], demonstrate important regional and temporal heterogeneity in outcomes among HIV-exposed infants. Consistent with global data on the scale-up of ARVs for vertical HIV prevention, particularly since 2010 [26], we found that uptake of maternal ARVs, including combination therapy regimens, was high in EA, CA, and SA, and has improved over time (trends were similar in other regions, though populations were smaller). Although infant receipt of ARV prophylaxis was suboptimal, there have been modest improvements over time. Also encouraging are our findings that 24-month cumulative incidences of HIV diagnosis and death among HIV-exposed infants were lower in more recent years, compared with the pre-2010 period.

While high maternal use of ARVs and ART during pregnancy and decreases in HIV diagnosis and death among HIV-exposed infants are reassuring, our findings raise concerns about other essential elements of PMTCT programs, particularly in CA and WA, where progress in preventing new HIV infections has lagged [27]. In contrast to EA and SA, where coverage of DNA PCR testing at 2 and 24 months of age was high and has remained high over time, early testing was suboptimal in both CA and WA, with little improvement in CA in recent years. While it is possible that more recent guidelines recommending rapid testing at older ages resulted in fewer DNA PCR tests in later infancy during recent periods, this may not be apparent in our population because most initial DNA PCR testing occurred before 18 months. In addition, the 24-month cumulative incidence of LTFU was high across all regions, and importantly, LTFU occurred early (<6 months of age) in most regions, contributing to gaps in infant prophylaxis and diagnostic testing. While our findings are consistent with prior literature [11,16–18], it is noteworthy that LTFU has not decreased in recent years in EA, WA, and SA, particularly as high LTFU contributes to the low percentages of infants documented as alive, in care, and HIV-free at 18 months noted across all regions and time periods.

Our findings should be viewed in the context of other data for the countries and regions included in this analysis. For example, while the regional heterogeneity in early HIV testing that we observed is consistent with regional differences reported elsewhere, our data show slightly higher uptake of DNA PCR testing compared with other regional estimates [3]—potentially reflecting better access to HIV care and laboratory monitoring for our study population than the broader population of HIV-exposed infants in these settings. Similarly, better access to ART and more effective treatment and prophylaxis regimens for our study population may have resulted in an observed 24-month cumulative incidence of HIV diagnosis in the most recent time period for EA (7·0%) that was slightly lower than 2015 and 2020 UNAIDS-modeled vertical transmission rates for Kenya, as well as an SA estimate (1·6%) substantially lower than modeled rates for Malawi and a CA estimate (2·9%) substantially lower than modeled rates for Burundi and DRC [3]. These differences, along with the lower transmission we noted in Malawi compared with concurrent studies from other SA settings [11,16], may also reflect resource disparities between clinical sites and variation in local epidemics. While observed mortality among our sample of HIV-exposed infants predictably exceeded rates documented among HIV-unexposed children [28], our survival estimates were similar to those from a pooled global analysis [14] and single-site studies [11,16], and our findings that mortality among HIV-exposed infants has decreased over time are novel.

For CA and EA, our multivariable analyses consistently supported the biologically expected associations between ART during pregnancy and decreased mortality and HIV transmission, as well as between LBW and death. Associations between later calendar time and decreased DNA PCR testing, HIV diagnosis, and death could reflect the increasing role of MCH clinics in the care of these infants in recent years; these peripheral sites may lag behind dedicated HIV care and treatment centers in their capacity for EID and subsequent DNA PCR testing [29], potentially leading to fewer HIV diagnoses. Such sites may also have less capacity for death ascertainment. Decreases in HIV diagnoses and deaths in recent years may reflect positive programmatic impacts of progressive expansions in treatment eligibility for women with HIV and their infants. In EA, observed associations of ART during pregnancy with increased DNA PCR testing and decreased LTFU are promising. However, alongside decreased DNA PCR testing and increased LTFU during later time periods in EA, these findings also suggest that infants of mothers not on ART may be at particular risk of care disengagement. LTFU in EA may be exacerbated by silent transfers [30] coincident with increasing decentralization of HIV care in the region over time.

To our knowledge, this is the largest analysis of HIV-exposed infants to date. Other notable study strengths include its geographical breadth, providing some of the most representative global information to date given that the dataset featured 4 African regions where the majority of women with HIV and their infants reside, and the inclusion of births occurring over a 25-year period. Although the long duration can complicate interpretation of results, including calendar time in multivariable models underscored some important trends in outcomes of interest, including reduced mortality and HIV diagnosis in later time periods, which may reflect advances in care for HIV-exposed infants (including the integration of HIV care into primary care), as well as secular trends in prevalence [2]. Another strength was our linkage between maternal and infant records, which allowed for the examination of associations between prenatal ART and infant mortality. It is worth noting that while ARVs were not universally available pre-2010, estimates were minimally impacted by the inclusion of data from years where there was little access to ARVs because our data had few infants born early in the study period (Table 1). For example, in the largest regional cohort (EA), 285/47,300 infants were born pre-2004, while in another large regional cohort (CA), 179/10,699 infants were born pre-2004.

Although our study included HIV-exposed infants from multiple geographical regions, constraints of the observational design and data preclude causal attribution of outcomes, and interpretation and comparisons should consider the precision of estimates. In addition, there are limitations to the generalization of our results to the global population of HIV-exposed infants, which is orders of magnitude larger and more widely distributed. Moreover, sites participating in IeDEA may not be broadly representative of program-specific strengths and deficiencies in some countries and regions. These limitations underscore the need to establish additional cohorts for the long-term follow-up of women with HIV and their children, built upon linked and standardized data collection, especially in understudied areas where progress in meeting global targets for prevention and treatment has stalled.

As data were collected in routine care settings, specific protocols for HIV testing, mortality ascertainment, visit frequency, and other processes likely varied by country, program, and calendar time, and there may be important differences between countries and programs that influenced our estimates. For example, if protocols for death ascertainment were less sophisticated in programs outside of EA, mortality estimates could be biased downwards relative to EA, with cumulative incidences of other outcomes biased upwards because death was a universal competing risk. Differences in data quality or consistency of collection could also bias results. Because laboratory testing is constrained by locally available resources, we defined HIV diagnosis based on a single positive result (i.e., not requiring substantiation by confirmatory testing). This may have resulted in some misclassification of this outcome, because a small proportion of positives (per a single test) are truly false positives that could have been identified as such by subsequent confirmatory testing. Similarly, misclassification of negative HIV status after 18 months was possible given that substantial numbers of infants are breastfed at 2 years of age [31]. This misclassification of HIV status (i.e., assigning an infant as negative when subsequent seroconversion was possible) could have biased our HIV diagnosis estimates downwards. Additionally, HIV diagnosis estimates may be impacted by the degree of HIV testing in a region, with increased diagnosis in EA where testing was near universal.

The exclusion of infants enrolled in sites that only provide care for those with confirmed HIV infection may have resulted in cumulative incidence underestimation in the case of HIV diagnosis and DNA PCR testing, or overestimation in the case of LTFU. Additionally, our decision to stratify results by categorical time periods defined by WHO eras of HIV treatment guidelines may have obscured some variation within time periods, and timely adoption of WHO recommendations may have differed across contributing programs, meaning that category boundaries may not have precisely reflected changes in practice. However, resultant impacts on estimates were likely mitigated by the multiyear duration of each period. Last, our dataset lacked the variables necessary to account for the influences of external factors such as forced migration, election violence, nursing strikes, and stock-outs of ARVs, cotrimoxazole, and laboratory test reagents that may disrupt care in resource-constrained areas.

These limitations notwithstanding, our findings reflect progress in the global scale-up of ARVs and ART for vertical HIV prevention since the early 2000s, while also providing insights about other key clinical and programmatic outcomes. They also elucidate ongoing vulnerabilities for HIV-exposed infants, particularly in CA and WA where progress has lagged, and for LBW infants who appear at markedly increased risk of death. Encouragingly, these vulnerabilities can be mitigated to varying degrees by addressing one consistent shortfall across regions: high LTFU, because better retention is logically tied to associated improvements in other identified areas of concern. Specifically, engagement in care facilitates the delivery of ARVs for HIV prevention, cotrimoxazole prophylaxis, breastfeeding and other nutritional support, and developmental interventions, all of which are reasonably expected to have downstream impacts on infectious comorbidity and mortality reduction. Even if shortfalls in testing access are identified and removed, only with improvements in consistent access to and uptake of quality and comprehensive care will we meet the goal of all exposed infants surviving and thriving [11], verified as free of HIV, throughout their childhoods and beyond.

## Supporting information

S1 Fig Cumulative incidences of DNA PCR testing, LTFU, HIV diagnosis, and death through 24 months of age among HIV-exposed infants in the EA region of the IeDEA consortium, by birth year, 2002–2020. While it would have been possible to include regions other than EA in S1 Fig, we opted not to because of sparser data and the potential to overinterpret year-specific results based on small numbers (for example, in CCASAnet, there were 1,795 infants in total across the time period), meaning that estimates for each year would have been derived from relatively few observations. In EA, higher LTFU in the early 2010s was potentially because PMTCT programs were being integrated into MCH clinics during this period, which may have resulted in some data capture issues. Higher deaths in the mid-2000s may be due to the introduction of formula feeding during this period, which may have increased deaths, as well as a larger proportion of infants who did not receive PMTCT services and entered care at older ages. As this was prior to the era of universal treatment, i.e., when ARV treatment initiation was based on immunologic criteria, more deaths may have occurred. Furthermore, in this period, pregnant women were not eligible for ARVs until 28 weeks of gestation, ARVs were typically discontinued within the first 2 months following delivery, and cotrimoxazole was not yet universally dispensed. ARV, antiretroviral; CCASAnet, Caribbean, Central, and South America network for HIV epidemiology; EA, East Africa; IeDEA, International epidemiology Databases to Evaluate AIDS; LTFU, loss to follow-up; MCH, maternal and child health; PMTCT, prevention of mother-to-child transmission.
(PDF)

S1 Table Cumulative incidences of DNA PCR testing, LTFU, HIV diagnosis, and death through 24 months of age (with and without HIV diagnosis as a competing risk) among HIV-exposed infants in the IeDEA consortium, by region, 1997–2020. Top estimates (before “/”) do not include HIV diagnosis as a competing risk; bottom estimates (after “/”) include this competing risk. HIV diagnosis cannot be a competing risk when it is the outcome. CL, confidence limit; IeDEA, International epidemiology Databases to Evaluate AIDS; LTFU, loss to follow-up.
(PDF)

S1 Concept Proposal Global analysis of key programmatic and clinical outcomes of HIV-exposed infants in the International epidemiology Databases to Evaluate AIDS consortium. (PDF)

S1 STROBE Checklist The RECORD statement—Checklist of items, extended from the STROBE statement, that should be reported in observational studies using routinely collected health data. (PDF)

S1 Text Acknowledgments. (PDF)

S1 Missingness Associations between variable missingness (risk ratios and 95% confidence intervals), and percentages of missing data by infant year of birth, in the EA and CA regions of the IeDEA consortium, 2001–2021. Reference category is other variable = nonmissing. *Example interpretation: Missingness of data on ART during pregnancy is associated with increased missingness of LBW data (risk ratio, 1·64; 95% confidence interval, 1·60–1·67). Note: Where blank, risk ratios were not calculable due to cells with zero counts (e.g., there were no records where LBW was missing but SGA was not missing). ART, antiretroviral therapy; CA, Central Africa; EA, East Africa; IeDEA, International epidemiology Databases to Evaluate AIDS; LBW, low birth weight; SGA, small for gestational age.
(PDF)

## Abbreviations

AP: Asia-Pacific
ART: antiretroviral therapy
ARV: antiretroviral
CA: Central Africa
CCASAnet: Caribbean, Central, and South America network for HIV epidemiology
DRC: Democratic Republic of Congo
EA: East Africa
EID: early infant diagnosis
IeDEA: International epidemiology Databases to Evaluate AIDS
IQR: interquartile range
LBW: low birth weight
LTFU: loss to follow-up
MCH: maternal and child health
NA-ACCORD: North American AIDS Cohort Collaboration on Research and Design
PMTCT: prevention of mother-to-child transmission
PTB: preterm birth
SA: Southern Africa
sdHR: subdistribution hazard ratio
SGA: small for gestational age
WA: West Africa
