# Supplementary material for: Clinical and programmatic outcomes of HIV-exposed infants enrolled in care at geographically diverse clinics, 1997–2021: A cohort study
Source: PLoS Med. 2022 Sep 15;19(9):e1004089. doi: 10.1371/journal.pmed.1004089 (PMC9477260; doi:10.1371/journal.pmed.1004089)
Supplement: S1 Fig — While it would have been possible to include regions other than EA in S1 Fig, we opted not to because of sparser data and the potential to overinterpret year-specific results based on small numbers (for example, in CCASAnet, there were 1,795 infants in total across the time period), meaning that estimates for each year would have been derived from relatively few observations. In EA, higher LTFU in the early 2010s was potentially because PMTCT programs were being integrated into MCH clinics during this period, which may have resulted in some data capture issues. Higher deaths in the mid-2000s may be due to the introduction of formula feeding during this period, which may have increased deaths, as well as a larger proportion of infants who did not receive PMTCT services and entered care at older ages. As this was prior to the era of universal treatment, i.e., when ARV treatment initiation was based on immunologic criteria, more deaths may have occurred. Furthermore, in this period, pregnant women were not eligible for ARVs until 28 weeks of gestation, ARVs were typically discontinued within the first 2 months following delivery, and cotrimoxazole was not yet universally dispensed. ARV, antiretroviral; CCASAnet, Caribbean, Central, and South America network for HIV epidemiology; EA, East Africa; IeDEA, International epidemiology Databases to Evaluate AIDS; LTFU, loss to follow-up; MCH, maternal and child health; PMTCT, prevention of mother-to-child transmission. (PDF) [file pmed.1004089.s001.pdf]

S1 Fig. Cumulative incidences of DNA PCR testing, loss to follow-up, HIV diagnosis, and death through 24 months of age among HIV-exposed infants in the East Africa region of the International epidemiology Databases to Evaluate AIDS consortium, by birth year, 2002-2020.

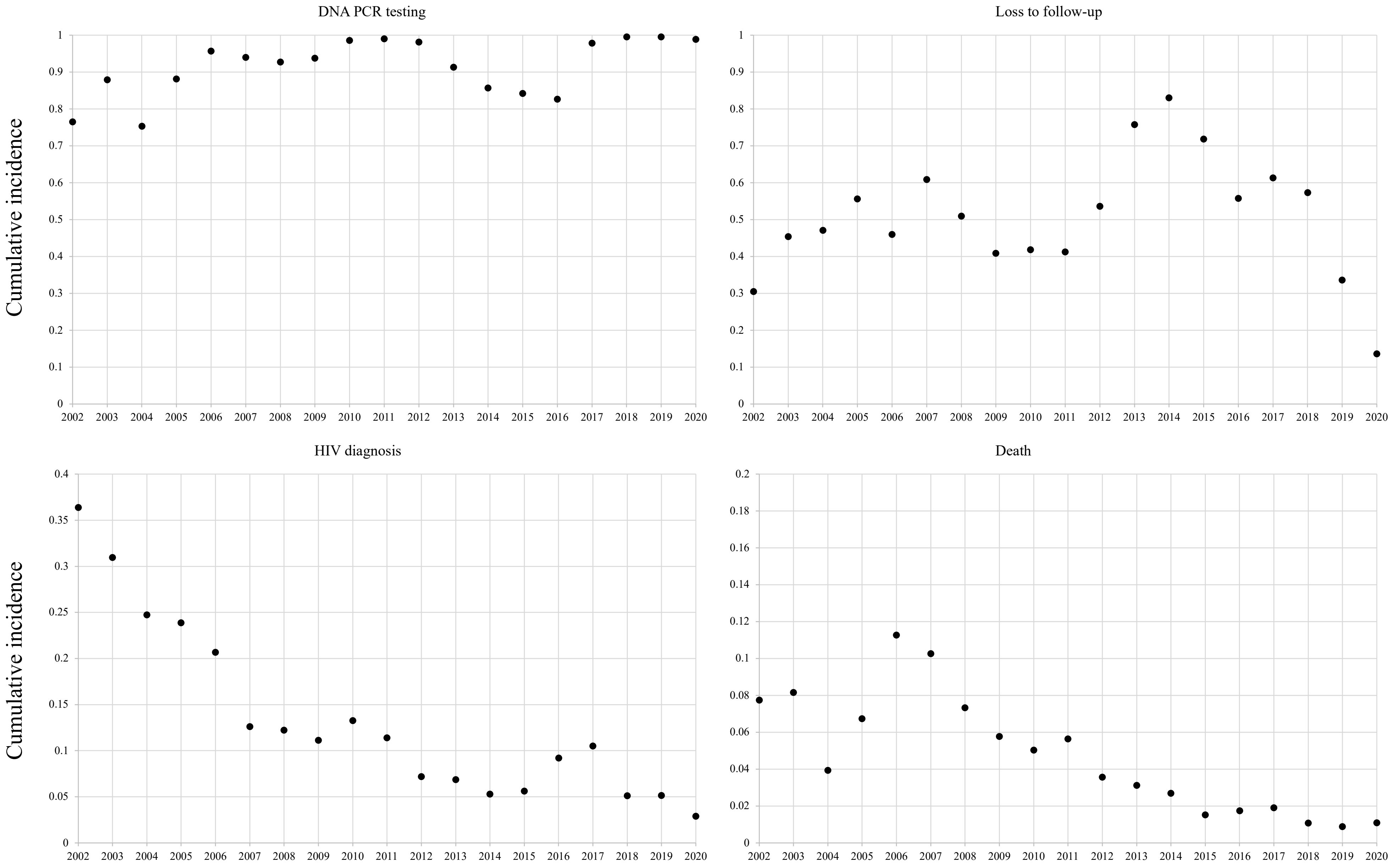

Note: While it would have been possible to include regions other than East Africa in S1 Fig, we opted not to because of sparser data and the potential to overinterpret year-specific results based on small numbers (for example, in CCASAnet, there were 1,795 infants in total across the time period), meaning that estimates for each year would have been derived from relatively few observations. In East Africa, higher loss to follow-up in the early 2010s was potentially because PMTCT programs were being integrated into MCH clinics during this period, which may have resulted in some data capture issues. Higher deaths in the mid-2000s may be due to the introduction of formula feeding during this period which may have increased deaths, as well as a larger proportion of infants who did not receive PMTCT services and entered care at older ages. As this was prior to the era of universal treatment, i.e., when antiretroviral treatment initiation was based on immunologic criteria, more deaths may have occurred. Furthermore, in this period, pregnant women were not eligible for antiretrovirals until 28 weeks of gestation, antiretrovirals were typically discontinued within the first two months following delivery, and cotrimoxazole was not yet universally dispensed.
