# Supplementary material for: Clinical and programmatic outcomes of HIV-exposed infants enrolled in care at geographically diverse clinics, 1997–2021: A cohort study
Source: PLoS Med. 2022 Sep 15;19(9):e1004089. doi: 10.1371/journal.pmed.1004089 (PMC9477260; doi:10.1371/journal.pmed.1004089)
Supplement: S1 Table — Top estimates (before “/”) do not include HIV diagnosis as a competing risk; bottom estimates (after “/”) include this competing risk. HIV diagnosis cannot be a competing risk when it is the outcome. CL, confidence limit; IeDEA, International epidemiology Databases to Evaluate AIDS; LTFU, loss to follow-up. (PDF) [file pmed.1004089.s002.pdf]

S1 Table. Cumulative incidences of DNA PCR testing, loss to follow-up, HIV diagnosis, and death through 24 months of age (with and without HIV diagnosis as a competing risk) among HIV-exposed infants in the International epidemiology Databases to Evaluate AIDS consortium, by region, 1997-2020.

	**East Africa**	**Central Africa**	**West Africa**	**Southern Africa**	**CCASAnet**
24-month cumulative incidence of DNA PCR testing (95% CL)	94.8% (94.6%-95.1%) / 91.8% (91.4%-92.2%)	60.1% (59.1%-61.1%) / 56.0% (54.9%-57.1%)	12.3% (11.2%-13.5%) / 11.8% (11.0%-12.8%)	90.2% (89.4%-91.0%) / 89.3% (88.4%-90.2%)	- / -
24-month cumulative incidence of loss to follow-up (95% CL)	56.2% (55.2%-57.1%) / 54.9% (53.9%-55.9%)	74.1% (73.3%-75.0%) / 73.1% (72.2%-73.9%)	98.5% (98.3%-98.7%) / 98.4% (98.2%-98.5%)	93.6% (92.9%-94.2%) / 93.3% (92.8%-93.8%)	65.5% (63.5%-67.5%) / 62.0% (59.8%-64.3%)
24-month cumulative incidence of HIV diagnosis (95% CL)	10.3% (9.7%-10.9%)	5.0% (4.5%-5.5%)	1.9% (1.6%-2.3%)	3.1% (2.7%-3.6%)	9.8% (8.6%-11.2%)
24-month cumulative incidence of death (95% CL)	4.7% (4.4%-5.0%) / 3.7% (3.4%-3.9%)	3.1% (2.8%-3.5%) / 2.6% (2.4%-3.0%)	1.9% (1.6%-2.4%) / 1.8% (1.4%-2.3%)	1.5% (1.1%-2.2%) / 1.4% (0.9%-2.2%)	0.5% (0.2%-1.0%) / 0.3% (0.1%-0.6%)

CL, confidence limit. Top estimates (before “/”) do not include HIV diagnosis as a competing risk; bottom estimates (after “/”) include this competing risk. HIV diagnosis cannot be a competing risk when it is the outcome.
