# Supplementary material for: Clinical and programmatic outcomes of HIV-exposed infants enrolled in care at geographically diverse clinics, 1997–2021: A cohort study
Source: PLoS Med. 2022 Sep 15;19(9):e1004089. doi: 10.1371/journal.pmed.1004089 (PMC9477260; doi:10.1371/journal.pmed.1004089)
Supplement: S1 Concept Proposal — (PDF) [file pmed.1004089.s003.pdf]

# CONCEPT SHEET FOR MULTI-REGIONAL ANALYSES

| | |
|---|---|
| **Date of EC approval:** | *28 March 2018* |
| **Tracking number:** | *MR118* |
| **Title:** | **Global analysis of key programmatic and clinical outcomes of HIV-exposed infants in the International epidemiology Databases to Evaluate AIDS consortium** |
| **Concept Lead:**<br>Email: | Andrew Edmonds:<br>aedmonds@email.unc.edu |
| **Collaborators:** | • Bev Musick, John Humphrey, Kara Wools-Kaloustian (East Africa)<br>• Mary-Ann Davies, Olivia Keiser, Malango Msukwa (Southern Africa and Umoyo+ Malawi)<br>• Valériane Leroy (West Africa)<br>• Adebola Adedimeji (Central Africa)<br>• Jimmy Carlucci, Marcelle Maia, Jorge Pinto (CCASAnet) |
| **IeDEA Correspondent:**<br>Email: | Andrew Edmonds:<br>aedmonds@email.unc.edu |
| **Data Manager:**<br>Email: | Andrew Edmonds:<br>aedmonds@email.unc.edu |
| **Lead Statistician:**<br>Email: | Andrew Edmonds:<br>aedmonds@email.unc.edu |
| **Where will data be merged?** | The University of North Carolina at Chapel Hill |
| **Where will statistical analyses be done?** | The University of North Carolina at Chapel Hill |
| **Abstract:**<br>(approximately 200 words) | **Background**<br>Despite continuing global increases in the uptake of antiretroviral (ARV) medications by mothers and infants to prevent vertical HIV transmission, various key issues related to HIV-exposed infants remain incompletely understood. These include receipt and timing of ARVs and HIV testing by this population, its |

| | |
|---|---|
| | susceptibilities to mortality and loss to follow-up (LTFU) from care, and trajectories of growth.<br><br>**Objectives**<br>Using a large multiregional dataset from the International epidemiology Databases to Evaluate AIDS, improving on the small sample size of prior single country/program studies of HIV-exposed infants, this project has three objectives focused on HIV-exposed infants:<br>1. Describe characteristics including preterm birth, low birth weight, small for gestational age, six-week underweight-for-age, ages at serological confirmation of infant HIV exposure and care enrollment, receipt of maternal/infant ARV prophylaxis and maternal antiretroviral therapy (ART), comorbidities, feeding modalities over the first two years of life (e.g., at six weeks and each three-month time point), other maternal factors such as age, etc.<br>2. Estimate the cumulative incidences of receipt of ARV prophylaxis and virological HIV testing, HIV infection, LTFU, and death through two years of age; estimate the cumulative incidence of ART initiation among infants identified as HIV-infected; estimate the associations between infant/maternal/facility characteristics and outcomes.<br>3. Analyze longitudinal measurements of weight, length, and head circumference to characterize growth evolution over the first two years of life.<br><br>**Methods**<br>1. Descriptive statistics – counts, percentages, medians and interquartile ranges, means and standard deviations, parametric/non-parametric statistical testing (e.g., chi square, Mann-Whitney, ANOVA), etc.<br>2. Estimation of subdistribution hazard ratios using multivariable Fine and Gray models, and estimation of cumulative incidence functions, each accounting for competing risks.<br>3. Mixed effect or generalized estimating equation models. |
| **Project outline:** (approximately 1000 words) | **Background**<br><br>In 2015, an estimated 1.2 million infants were born to women living with HIV in the 21 Global Plan priority countries.[1] Although increasing numbers of mothers and infants worldwide are accessing the antiretroviral (ARV) medications necessary to reduce vertical HIV transmission during the perinatal and postnatal periods,[1] there are still limited data from low- and middle-income countries on several key indicators. Items in need of further description include the timing of delivery of prophylactic ARVs and HIV testing to HIV-exposed infants, as well as this population's susceptibility to early mortality and loss to follow-up (LTFU) from care, itself a driver of HIV incidence and mortality. Additionally, while accumulating evidence suggests that |

| | |
|---|---|
| | exposures to HIV and ARV medications in infants exposed to HIV may result in deficiencies in birthweight as well as growth during early childhood,[2,3] which both may lead to developmental delays, morbidity, and mortality, patterns and predictors of growth among HIV-exposed children remain incompletely characterized.<br><br>While characteristics and outcomes of HIV-exposed infants have been examined in various prior investigations, these studies have generally been limited to relatively small populations in single-country programs. For example, in the Democratic Republic of Congo, HIV testing and transmission, ARV treatment (ART) and prophylaxis, mortality, and LTFU were evaluated in fewer than 2,000 infants.[4-6] Prevalence of preterm delivery, low birth weight (LBW), small for gestational age (SGA), and six-week underweight-for-age (UFA), as well as the associations of these outcomes with maternal ARV exposure, were appraised in a study of 2,500 infants in South Africa.[7] In 4,000 HIV-exposed infants in Zimbabwe, head circumference over the first year of life was impaired compared to a comparator group of HIV-unexposed infants.[8] In a larger recent study of over 11,000 infants in Malawi, investigators estimated that while HIV mortality and transmission were low, a majority of the population was LTFU leading to inadequate HIV diagnosis and ART initiation.[9]<br><br>Five regions of the IeDEA consortium, Southern Africa, Central Africa, East Africa, West Africa, and the Caribbean, Central and South America network (CCASAnet), collect data on HIV-exposed infants, providing a unique opportunity to explore and provide needed evidence on vital questions related to this understudied population. Through the analysis of a large, multiregional pooled dataset from five IeDEA regions, this project will provide generalizable, robust information not often attainable from prior studies of smaller HIV-exposed infant populations, and will identify regions and points in the care continuum that should be targeted with interventions to strengthen service delivery. We will complete three objectives focused on key programmatic and clinical outcomes of HIV-exposed infants:<br><br>**Objectives**<br><br>1. Provide a descriptive overview of the characteristics of IeDEA HIV-exposed infant populations, including items such as preterm birth, LBW, SGA, six-week UFA, ages at serological confirmation of infant HIV exposure and care enrollment, receipt of maternal/infant ARV prophylaxis and maternal ART, comorbidities, feeding modalities (e.g., exclusive breastfeeding, mixed feeding, introduction of solids, etc., at six weeks and each three-month time point), other maternal factors such as age, etc.<br><br>2. Among these HIV-exposed infants, estimate the cumulative incidences of receipt of ARV prophylaxis and |

virological HIV testing, HIV infection (an approximation of vertical transmission rate), LTFU, and death through two years of age, as well as the cumulative incidence of ART initiation among infants identified as HIV-infected; estimate the associations between infant/maternal/facility characteristics and outcomes.

3. Using longitudinal measurements of weight, length, and head circumference, characterize the growth evolution of HIV-exposed infants over the first two years of life.

For each objective, data will be examined within and across 1) regions, and 2) calendar time periods, with temporal trends within regions, and site variability, evaluated whenever possible.

**Data availability**

Key variables for the proposed objectives include HIV testing dates/types/outcomes, receipt of maternal/infant ARV prophylaxis and ART, visit/birth/death dates, longitudinal length and weight measurements, and documentation of birth to an HIV-infected mother. While these are generally available in all regions during all time periods, other variables are only available in specific regions and time periods. As such, items with limited availability (e.g., infant HIV antibody testing, feeding modalities, comorbidities) will be evaluated to the extent that data permit.

The following table summarizes counts and birth years of HIV-exposed infants in IeDEA databases, by region and country.

| IeDEA region | Country | Number | Birth years |
|---|---|---|---|
| East Africa | Kenya | 36,000 | 2002-2017 |
| Southern Africa | Malawi | >16,200 | 2009-2015 |
| | South Africa | 2,600 | To be determined |
| West Africa | Cote d'Ivoire, Togo, Benin, Ghana | 5,100 (across region) | 2002-2017 (across region) |
| Central Africa | Democratic Republic of Congo | 2,200 | 2007-2013 |
| | Rwanda / Burundi | To be determined | To be determined |
| CCASAnet | Brazil | 2,300 | 1991-2017 |
| **Total** | | **~65,000** | **1991-2017** |

**Definitions**

- LTFU: no visit for 12 months; follow-up censored on date of last visit. Alternative definitions of LTFU (e.g., no visit for 3 or 6 months) will also be explored.
- Death: Documentation of date of death in database.
- Preterm birth: birth at fewer than 37 weeks of gestational age. LBW: birthweight of < 2500 grams. SGA: birthweight below the 10$^{th}$ percentile for babies of the same gestational age.[10]
- Weight-for-age z-score (WAZ), length-for-age z-score (LAZ), and head circumference-for-age z-score (HCAZ) will based on WHO standards.[11] UFA: WAZ < -2.
- HIV infection: HIV virological test (e.g., DNA PCR, RNA viral load) positive at any age, or serological test positive at ≥ 18 months of age.

**Eligibility criteria**

Infants enrolled in care at less than 18 months of age with either 1) documentation of birth to an HIV-infected mother, or 2) serological evidence of HIV exposure, will be eligible for these analyses. Serological evidence of HIV exposure will not be required for inclusion because the anticipated extent of missing data would substantially decrease the available sample size, i.e., documentation of birth to an HIV-infected mother alone is sufficient. All IeDEA infants meeting either of these criteria, regardless of calendar year of birth/enrollment or whether they transferred in from another facility, will be eligible for inclusion in this study. There will not be an HIV-unexposed infant comparator group for any analysis, as there are no such data in IeDEA.

**Statistical analyses**

For Objective 1, we will describe and compare characteristics and outcomes of HIV-exposed infant populations using appropriate metrics; e.g., counts, percentages, medians and interquartile ranges, means and standard deviations, parametric/non-parametric tests (e.g., chi square, Mann-Whitney, ANOVA), etc.

For Objective 2, we will estimate the cumulative incidences of the specified outcomes accounting for competing risks, and compare strata/subgroups (e.g., maternal age, clinical/virological/immunological characteristics, receipt of ART or ARV prophylaxis; infant WAZ, enrollment age, ARV prophylaxis, feeding modality; calendar year; facility type; etc.) using Gray's test for equality.[12] To characterize associations between the above factors and the outcomes, subdistribution hazard ratios will be estimated using multivariable Fine and Gray

| | |
|---|---|
| | models accounting for competing risks.[13] We will employ non-parametric estimation of cumulative incidence functions incorporating the cause-specific cumulative hazard function estimated by the Nelson-Aalen estimator and the survival function estimated from the Kaplan-Meier estimator;[14] competing risks such as death (for the LTFU outcome) and LTFU (for the death outcome) will be appropriately considered, as will the sensitivity of results to treatment of LTFU as a censoring event vs. as a competing event.[15] Use of both the age and time since enrollment time scales will be explored.<br><br>For Objective 3, methods such as mixed effect[16-17] (with appropriate transformations) or generalized estimating equations models[18-19] will be used to characterize growth evolution in the population, as well as impacts of the factors outlined in Objective 2 above. Outcomes to be examined will include HCAZ, WAZ, and LAZ, and descriptive statistics (such as medians and interquartile ranges) will be used to summarize parameters at developmentally relevant time points. Covariates to be considered for this objective include those outlined in Objective 2 above, and will be included if there is sufficient evidence in the literature of meaningful associations with the growth outcomes.<br><br>In all aims, primary analyses will include all HIV-exposed infants, even those later determined to be HIV-infected. Subsequent sensitivity analyses will be restricted to infants with 1) ruled-out HIV infection (in non-breastfed infants, 2 or more negative virological tests, with 1 obtained at age ≥ 1 month and 1 at age ≥4 months, or 2 negative HIV antibody tests from separate specimens obtained at age ≥ 6 months) or yet undetermined HIV infection status (lack of positive virological test, and not ruled-out as defined above), and 2) confirmed HIV-uninfected status (as defined above). This approach will provide specific information not just on outcomes among infants exposed to (but not infected with) HIV, but also among the globally relevant population of all HIV-exposed infants, some of whom become infected with HIV in their first years of life. We will also explore the sensitivity of our results to alternative definitions of LTFU (e.g., no visit for 3 or 6 months) as there is currently no standard in the literature.<br><br>Standard software packages and will be used for all analyses (e.g., %CIF[14] and %PSHREG[20] macros in SAS, SAS Proc GENMOD and GLIMMIX, WHO Anthro SAS macro,[21] etc.) |
| **References:** | 1. UNAIDS. On the Fast-track to an AIDS-Free Generation. 2016.<br>2. Evans C, Jones CE, Prendergast AJ. HIV-exposed, uninfected infants: new global challenges in the era of paediatric HIV elimination. Lancet Infect Dis. 2016 Jun;16(6):e92-e107.<br>3. Sugandhi N, Rodrigues J, Kim M, Ahmed S, Amzel A, Tolle M, Dziuban EJ, Kellerman SE, Rivadeneira E; Child Survival Working Group of the Interagency Task Team on the Prevention and Treatment of HIV infection in Pregnant Women, Mothers and |

Children. HIV-exposed infants: rethinking care for a lifelong condition. AIDS. 2013 Nov;27 Suppl 2:S187-95.

4. Edmonds A, Feinstein L, Okitolonda V, Thompson D, Kawende B, Behets F. Implementation and Operational Research: Decentralization Does Not Assure Optimal Delivery of PMTCT and HIV-Exposed Infant Services in a Low Prevalence Setting. J Acquir Immune Defic Syndr. 2015 Dec 1;70(4):e130-9.

5. Feinstein L, Edmonds A, Chalachala JL, Okitolonda V, Lusiama J, Van Rie A, Chi BH, Cole SR, Behets F. Temporal changes in the outcomes of HIV-exposed infants in Kinshasa, Democratic Republic of Congo during a period of rapidly evolving guidelines for care (2007-2013). AIDS. 2014 Jul;28 Suppl 3:S301-11.

6. Feinstein L, Edmonds A, Okitolonda V, Cole SR, Van Rie A, Chi BH, Ndjibu P, Lusiama J, Chalachala JL, Behets F. Implementation and Operational Research: Maternal Combination Antiretroviral Therapy Is Associated With Improved Retention of HIV-Exposed Infants in Kinshasa, Democratic Republic of Congo. J Acquir Immune Defic Syndr. 2015 Jul 1;69(3):e93-9.

7. Ramokolo V, Goga AE, Lombard C, Doherty T, Jackson DJ, Engebretsen IM. In Utero ART Exposure and Birth and Early Growth Outcomes Among HIV-Exposed Uninfected Infants Attending Immunization Services: Results From National PMTCT Surveillance, South Africa. Open Forum Infect Dis. 2017 Aug 30;4(4):ofx187.

8. Evans C, Chasekwa B, Ntozini R, Humphrey JH, Prendergast AJ. Head circumferences of children born to HIV-infected and HIV-uninfected mothers in Zimbabwe during the preantiretroviral therapy era. AIDS. 2016 Sep 24;30(15):2323-8.

9. Haas AD, van Oosterhout JJ, Tenthani L, Jahn A, Zwahlen M, Msukwa MT, Davies MA, Tal K, Phiri N, Spoerri A, Chimbwandira F, Egger M, Keiser O. HIV transmission and retention in care among HIV-exposed children enrolled in Malawi's prevention of mother-to-child transmission programme. J Int AIDS Soc. 2017 Sep 4;20(1):21947.

10. Fenton TR, Kim JH. A systematic review and meta-analysis to revise the Fenton growth chart for preterm infants. BMC Pediatr. 2013 Apr 20;13:59.

11. The WHO Child Growth Standards. Available at: http://www.who.int/childgrowth/standards/en/

12. Gray RJ: A class of K-sample tests for comparing the cumulative incidence of a competing risk. Ann Stat 16:1141-1154, 1988.

13. Fine JP, Gray RJ: A proportional hazards model for the subdistribution of a competing risk. J Am Stat Assoc 94:496-509, 1999.

14. Lin G, So Y, Johnston G. Analyzing Survival Data with Competing Risks Using SAS Software. Proceedings of the SAS Global Forum 2012 Conference, Cary, NC: SAS Institute Inc.

| | |
|---|---|
| | 15. Strassle PD, Rudolph JE, Harrington BJ, Levintow SN. Lost Opportunities Concerning Loss-to-Follow-up: A Response to Elul et al. J Acquir Immune Defic Syndr. 2017 Jun 1;75(2):e55-e56.<br>16. Checkley W, Epstein LD, Gilman RH, Cabrera L, Black RE. Effects of acute diarrhea on linear growth in Peruvian children. Am J Epidemiol. 2003 Jan 15;157(2):166-75.<br>17. Chirwa ED, Griffiths PL, Maleta K, Norris SA, Cameron N. Multi-level modelling of longitudinal child growth data from the Birth-to-Twenty Cohort: a comparison of growth models. Ann Hum Biol. 2014 Mar-Apr;41(2):168-79.<br>18. Assis AM, Barreto ML, Santos LM, Fiaccone R, da Silva Gomes GS. Growth faltering in childhood related to diarrhea: a longitudinal community based study. Eur J Clin Nutr. 2005 Nov;59(11):1317-23.<br>19. Eckhardt CL, Suchindran C, Gordon-Larsen P, Adair LS. The association between diet and height in the postinfancy period changes with age and socioeconomicstatus in Filipino youths. J Nutr. 2005 Sep;135(9):2192-8.<br>20. Kohl M, Plischke M, Leffondré K, Heinze G. PSHREG: a SAS macro for proportional and nonproportional subdistribution hazards regression. Comput Methods Programs Biomed. 2015 Feb;118(2):218-33.<br>21. WHO Anthro and macros. Available at: http://www.who.int/childgrowth/software/en/ |
| **Ethics:** | *Select as appropriate:*<br>☒ This concept uses only the IeDEA standard dataset and is covered by the core IeDEA ethics approvals.<br>☐ This concept requires additional collection of health-related data, measurements or tests, or sampling of biological material not included in the IeDEA standard dataset. Additional ethics approval is required. |
| **Target journal(s):** | JAIDS, AIDS, JIAS, JPIDS, PIDJ |
| **Milestones:** | Circulation of concept sheet to Executive Committee: 2/2018<br>Approval of concept sheet by Executive Committee: 3/2018<br>Preparation of data and transfer: 4-6/2018<br>Analysis: 7-10/2018<br>Approval of analysis report by Executive Committee: 12/2018<br>Paper submitted to journal: 2019 |
