# Supplementary material for: Clinical and programmatic outcomes of HIV-exposed infants enrolled in care at geographically diverse clinics, 1997–2021: A cohort study
Source: PLoS Med. 2022 Sep 15;19(9):e1004089. doi: 10.1371/journal.pmed.1004089 (PMC9477260; doi:10.1371/journal.pmed.1004089)
Supplement: S1 Missingness — Reference category is other variable = nonmissing. *Example interpretation: Missingness of data on ART during pregnancy is associated with increased missingness of LBW data (risk ratio, 1·64; 95% confidence interval, 1·60–1·67). Note: Where blank, risk ratios were not calculable due to cells with zero counts (e.g., there were no records where LBW was missing but SGA was not missing). ART, antiretroviral therapy; CA, Central Africa; EA, East Africa; IeDEA, International epidemiology Databases to Evaluate AIDS; LBW, low birth weight; SGA, small for gestational age. (PDF) [file pmed.1004089.s006.pdf]

S1 Missingness. Associations between variable missingness (risk ratios and 95% confidence intervals), and percentages of missing data by infant year of birth, in the East Africa and Central Africa regions of the International epidemiology Databases to Evaluate AIDS consortium, 2001-2021.

						Infant year of birth			
		Antiretroviral therapy during pregnancy	Low birth weight	Preterm birth	Small for gestational age	pre-2010	2010-2013	2014-2015	post-2015
**East Africa**									
	Antiretroviral therapy during pregnancy	-	1·58 (1·56-1·61)	2561 (1480-4432)		50·7%	38·9%	33·7%	31·1%
Maternal or infant characteristic	Low birth weight	1·64 (1·60-1·67)*	-	1·60 (1·57-1·63)		47·5%	35·2%	25·9%	33·0%
	Preterm birth	12·1 (11·8-12·6)	1·49 (1·46-1·51)	-		54·8%	43·0%	40·6%	36·1%
	Small for gestational age	2·84 (2·81-2·88)	2·61 (2·57-2·64)	3·40 (3·35-3·46)	-	71·2%	58·7%	53·6%	55·4%
**Central Africa**									
	Antiretroviral therapy during pregnancy	-	2·91 (2·67-3·17)	4·39 (4·07-4·73)	2·52 (2·33-2·73)	29·6%	21·4%	7·7%	5·9%
Maternal or infant characteristic	Low birth weight	3·22 (2·91-3·56)	-	4·95 (4·50-5·44)		15·1%	7·9%	8·1%	8·3%
	Preterm birth	4·72 (4·35-5·12)	4·46 (4·10-4·85)	-		15·9%	12·4%	9·4%	10·9%
	Small for gestational age	2·15 (2·03-2·28)	6·43 (6·19-6·69)	7·61 (7·28-7·95)	-	24·7%	20·3%	20·4%	25·7%

Reference category is other variable = non-missing. *Example interpretation: Missingness of data on antiretroviral therapy during pregnancy is associated with increased missingness of low birth weight data (risk ratio, 1·64; 95% confidence interval, 1·60-1·67).

Note: Where blank, risk ratios were not calculable due to cells with zero counts (e.g., there were no records where low birth weight was missing but small for gestational age was not missing).
